# Optimized protocol for whole organ decellularization

**DOI:** 10.1186/s40001-017-0272-y

**Published:** 2017-09-08

**Authors:** A. Schmitt, R. Csiki, A. Tron, B. Saldamli, J. Tübel, K. Florian, S. Siebenlist, E. Balmayor, R. Burgkart

**Affiliations:** 1 Department of Sports Orthopedics, Klinikum rechts der Isar der Technischen Universität München, Munich, Germany; 2 Department of Orthopedics, Klinikum rechts der Isar der Technischen Universität München, Munich, Germany; 3 Department of Experimental Traumatology, Klinikum rechts der Isar der Technischen Universität München, Munich, Germany

**Keywords:** Tissue engineering, Vascularization, Decellularization

## Abstract

**Background:**

The idea of tissue decellularization to gain matrices for tissue engineering is promising. The aim of the present study is to establish a safe and reproducible protocol for solid tissue decellularization that prevents the architecture of the matrix with the inherent vascular network.

**Methods:**

The study was performed in rat kidneys which were decellularized by a SDS-based perfusion protocol. Perfusion time and SDS concentration were systematically changed to obtain the shortest and most gentle protocol that leads to complete decellularization.

**Results:**

We investigated kinetics of protein elution, decellularization success, and remaining cell toxicity. This resulted in a reproducible protocol, leading to safe decellularization with prevention of the inherent vascular network, without remaining detectable cell toxicity. The established protocol leads to solid tissue decellularization in only 7 h, which is by far shorter than the previously published methods.

**Conclusion:**

The established technique has the potential to become a relevant platform technology for tissue engineering of solid tissues. It provides a solution for the yet-unsolved problem of vascularization.

## Background

Nowadays, great hope is given to tissue engineering techniques to create new tissues, or even whole organs. Even if there has been substantial progress in this research field during the recent years, tissue engineering procedures have barely reached the patient.

The main problem in tissue engineering of solid tissues remains the vascularization of the constructs [1]. The lack of vascularization will lead to necrosis of tissue samples larger than 400 µm in diameter [2].

The use of decellularized tissues or organs as tissue engineering matrix could provide a solution for this challenge. A proper decellularization without destruction of the vascular network could provide a tissue engineering matrix with inherent vascularity. During the recent years, researchers have seized the idea of decellularization. This resulted in some breakthroughs in the whole organ tissue engineering. In 2008, a perfused, pumping heart could be generated in vitro by reseeding a decellularized rat heart with cardiomyocytes [3]. Furthermore, recellularized liver matrix could be successfully transplanted into a rat model [4]. Even whole lungs were successfully decellularized, recolonialized, and replanted in a rat model, where they provided gas exchange in vivo [5, 6].

Recently, our group could extend the relevance of the decellularization technique as platform technology for tissue engineering. We could give a proof of principle that the decellularization technique can provide a universal matrix for tissue engineering, even beyond tissue and species barriers [7].

The aim of the present study is to optimize the decellularization protocol for solid organs. Decellularization parameters and washing steps will be systematically investigated to reach an optimized, fast, and reproducible procedure that prevents the architecture of the extracellular matrix without leaving toxic remnants and cell residues.

## Methods

### Decellularization of rat kidneys

Rat kidneys were harvested from cadavers of approximately 500 g Sprague-Dawley rats. Kidneys were obtained from the animals within 30 min after euthanasia and frozen at −80 °C in PBS for 4–12 weeks. There was no anticoagulation medication applied to the animals before harvesting of the kidneys. For decellularization procedure, the kidneys were thawed, the surrounding soft tissue was removed, and a cannula was fixed in the renal artery. That cannula was then connected to a pressure-controlled roller pump (Arthrex AR-6475).

The decellularization procedure was performed at ambient temperature at a perfusion pressure of 100 mmHg. The decellularization procedure was performed at sterile conditions and contains multiple steps as illustrated in Table 1.

**Table 1:** Steps of the decellularization procedure

Step	Perfusion solution	Volume (ml)	Perfusion time
1	PBS	200	10 min
2	SDS (concentration variable, compare Table 2)	300	Variable (compare Table 2)
3	PBS (1st washing step)	200	60 min
4	PBS (2nd washing step)	200	60 min
5	PBS (3rd washing step)	200	60 min
6	PBS (4th washing step)	200	60 min
7	PBS (5th washing step)	200	60 min

### Protein detection using BCA assay

Total protein content in flowout from the washing steps was determined using BCA Protein Assay Kit (Thermo Scientific), according to the manufacturer’s protocol.

### Histology

For histologic investigations, samples were embedded into paraffin, cut into 3-µm slices, deparaffinized, and rehydrated. Samples were stained with hematoxylin and eosin. In addition, immunohistochemical stainings were performed. For immunohistochemistry, endogenous peroxidase was inactivated, and immunohistochemistry was performed using the Vectastain ABC avidin–biotin system (Vector Laboratories) in combination with AEC substrate chromogen (Dako). Primary antibodies were used in the following concentrations: anti-Laminin 1:500 (Dako), anti-Fibronectin 1:250 (Dako). Isotype anti-IgG anti-bodies were used as negative controls in corresponding dilutions. Counterstaining was performed using hematoxylin.

For SYBR Green staining, the SYBR Green I reagent was used at a concentration of 1:10,000 in PBS on paraformaldehyde-fixed paraffin- embedded tissue slices.

### Allura red staining

10 mg Allura Red (Sigma) was added to 20 mL of freshly made liquid solution of porcine gelatin in warm distilled water. The prepared solution was injected into the renal artery after decellularization.

### SDS detection

For direct detection of SDS within the washing solutions, we used the method described by Rusconi et al. [8]. In brief, 10 µl washing solution was mixed with 150 µl reagent solution (1 ml stains-all standard solution [2 mg Stains-All, 1 ml isopropanol, 1 ml dH_2_O], 1 ml formamide, 18 ml dH_2_0). Photometric detection was performed at 450 nm and quantified using a SDS standard curve. Validation of the method revealed a detection limit of 0.0025% SDS in our experimental setting.

### Cell culture

Primary human osteoblasts were isolated from femur heads of patients undergoing prosthetic replacement, as previously described [7]. This happened in accordance with the ethical code of ‘‘Klinikum rechts der Isar’’ (Technical University Munich, Germany) and having obtained the patients’ written consent. Osteoblasts were cultivated in DMEM with 1 g/l glucose, supplemented with 15% FCS, 1% l-glutamine, 1% MEM vitamins, 2% HEPES buffer, 100 U/ml penicillin, 100 mg/ml streptomycin, and 285 mM l-ascorbate-2-phosphate, whereas C2C12-cells were cultivated in DMEM with 4.5 g/L glucose, supplemented with 10% FCS, 1% l-glutamine, 1% MEM vitamins, 2% HEPES buffer, 100 U/ml penicillin, 100 mg/ml streptomycin. The medium was changed every 5 days. Primary osteoblasts were used for experiments in the 3rd–5th passages. Before experimental use, the phenotype of primary osteoblasts was verified by alkaline phosphatase (ALP), collagen type I, and osteocalcin staining.

### Alamar blue and LDH assays

Alamar blue assay (AbD Serotec) and Cytotoxicity Detection Kit (LDH) (Roche) were used according to the manufacturer’s protocol. For toxicity testing, C2C12-cells were seeded at a density of 15,000 cells/cm^2^. Adherent cells were incubated for 24 h with a 1:1 mixture of culture medium and the washing solution to test. As controls, a 1:1 mixture of culture medium and 0.1% Triton X-100, respectively, of 0.05% and 0.005% SDS was used. After 24 h, the test medium was probed for LDH, and cells were incubated with the Alamar blue reagent for 3 h. In Alamar blue testing, cells cultivated with fully supplemented cell culture medium (DMEM) were used as positive control. All other groups were drafted as “relative survival” according to the DMEM group. For LDH testing, 0.1% Triton X-100 was used as positive control. This concentration leads to full death of the indicator cells. All other groups were drafted as “relative survival” according to the 0.1% Triton X-100 group.

### Seeding and culture of cells in decellularized rat kidneys

Cells were seeded through the vascular system into the decellularized kidneys through the attached catheter. 1 × 10^7^ cells were suspended in 10 ml culture medium and injected into the renal artery. The seeded kidney scaffolds were placed in sterile flasks in a cell culture incubator under normal cell culture conditions (37 °C, 5% CO_2_). Perfusion under 50 mmHg pressure was started 4 h after seeding with osteoblast growth medium (DMEM w/o calcium, supplemented with 15% FCS, 1% l-glutamine, 2% HEPES buffer, 100 U/ml penicillin, 100 mg/ml streptomycin, 285 mM l-ascorbate-2-phosphate, and 100 nM dexamethasone). At 24 h after seeding, the perfusion pressure was increased to 100 mmHg, and a total of 300 ml of cell culture medium was recirculated. Medium was changed every 3 days.

## Results

### Decellularization kinetics

**Fig. 1:**
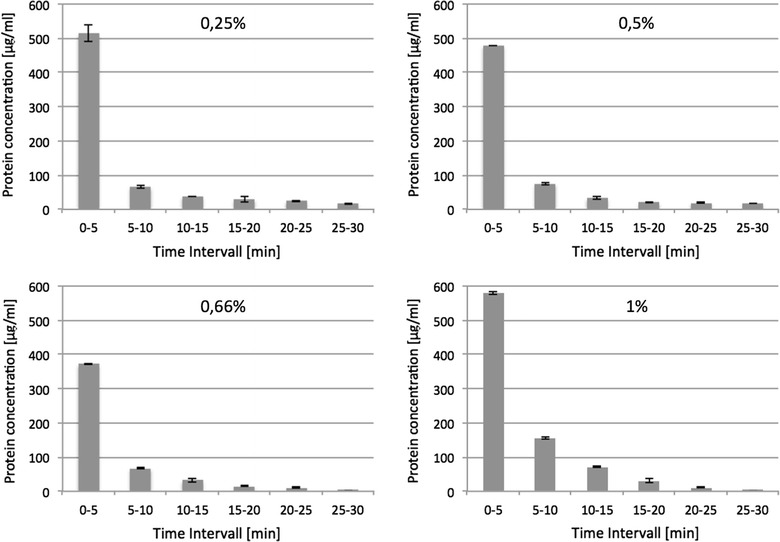
Protein elution kinetics during the decellularization process. Native rat kidneys were perfused with different concentrations of SDS solution. Flowout of SDS solution from kidneys was collected at 5-min intervals and investigated for protein content using BCA assay. Most protein is eluted within the first 5 min of perfusion. Protein levels are decreasing over time. After 30 min of perfusion, detectable protein concentrations are still eluted from the tissue. *n* = 1

In the first step, the decellularization process was further characterized. Therefore, native rat kidneys were perfused with SDS solution of different concentrations (0.25, 0.5, 0.66, and 1%). The outflow from the kidney was collected at 5-minute intervals and investigated for protein content. Most protein was eluted within the first 5 min of the decellularization process. Over the subsequent periods, protein concentration within the perfusion solution continuously decreased. However, even after 30 min, protein was still eluted from the kidneys (Fig. 1).

### Decellularization efficacy of different protocols

For systematic investigation of decellularization efficacy with different SDS concentrations and perfusion times, native rat kidneys were perfused with different combinations of both parameters. These combinations are shown in Table 2. Three kidneys were decellularized for each combination. Success of decellularization was confirmed macroscopically and by histology (HE Staining).

**Table 2:** Overview over the decellularization efficacy of the different protocols

Perfusion SDS concentration (%)	30 min	60 min	120 min
0.25	**✗**	**✗**	**✓**
0.50	**✗**	**✗**	**✓**
0.66	**✓**	**✓**	**✓**
1.00	**✗**	**✓**	**✓**

Three rat kidneys were decellularized for each condition (Perfusion time/SDS concentration). Success of decellularization was investigated macroscopically and with histology (HE-Staining). **✓:** complete decellularization of all three kidneys; **x:** Detectable cell residues in at least one kidney. Perfusion for at least 60 min with at least 0.66% SDS results in reliable decellularization. *n* = 3

**Fig. 2:**
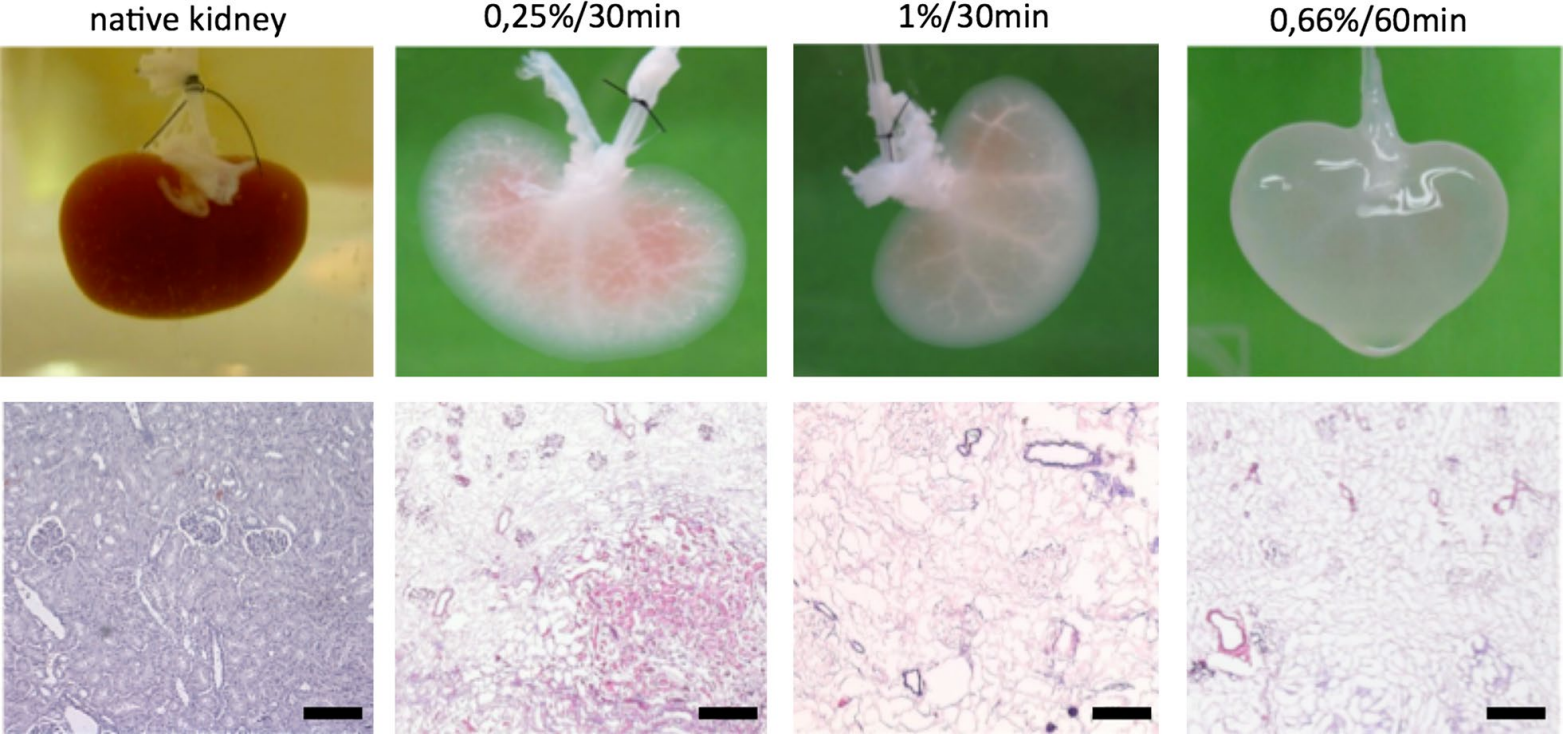
Macroscopic and histologic investigation of decellularization efficacy of different SDS concentration/perfusion time combinations.* Upper row* representative macroscopic pictures of the kidneys.* Lower row* representative HE-Staining. 30-min perfusion leads to insufficient decellularization with macroscopic and microscopic detectable cell remnants. 60-min perfusion with 0.66% SDS leads to full decellularization in all three treated kidneys. Representative pictures. Magnification 10×. *Black bars* indicate 200 µm. *n* = 3

Perfusion for 30 min did not result in reliable and sufficient decellularization. Even after perfusion with 1% SDS, persisting cells were detectable in one of three kidneys. In contrast, perfusion for 60 min with SDS concentrations of 0.66% and more resulted in complete and reproducible decellularization in all three kidneys. After 120-min perfusion, even the lower SDS concentrations led to complete decellularization in all three samples (Table 2; Fig. 2).

### Preservation of matrix

**Fig. 3:**
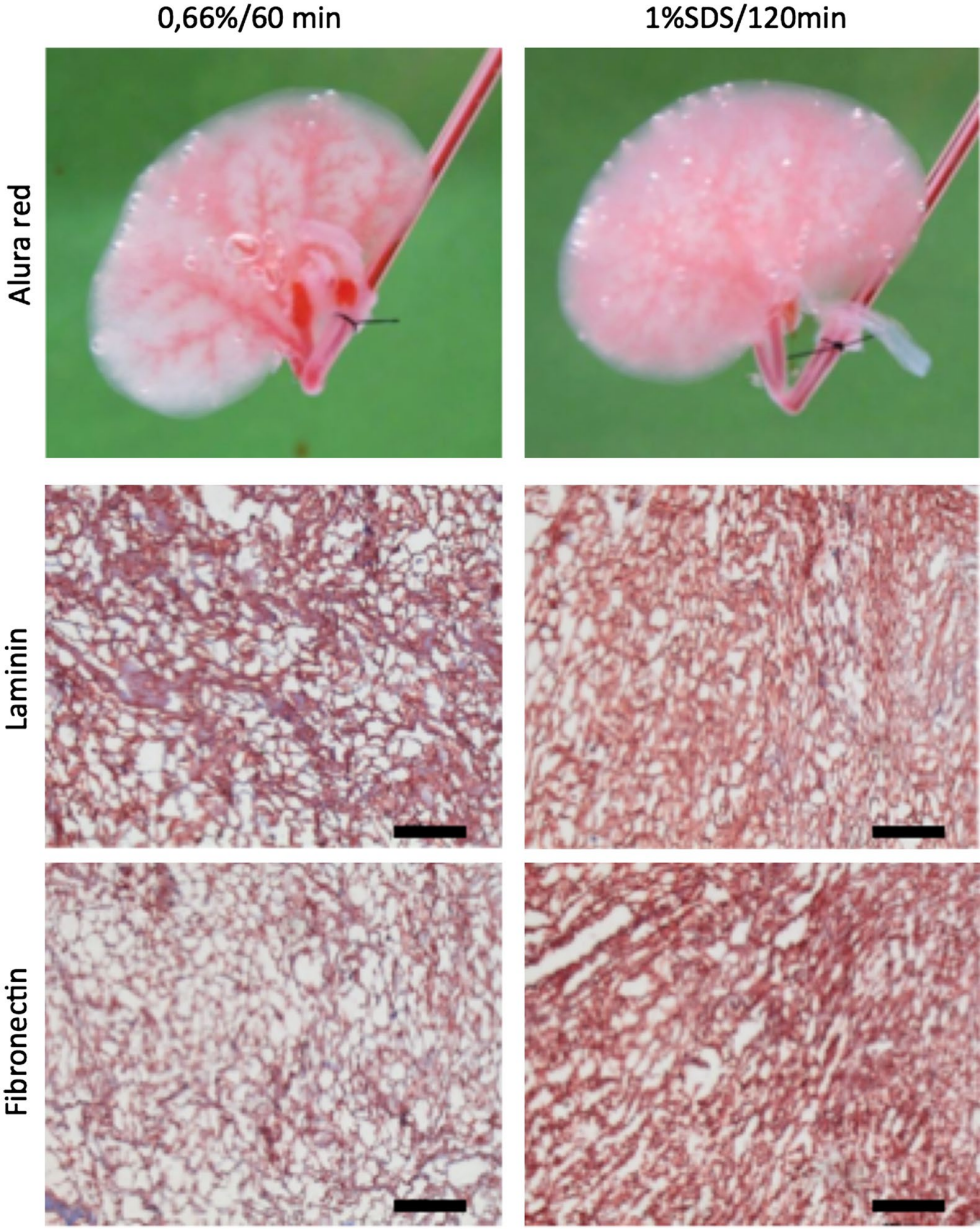
Integrity of the extracellular matrix after decellularization procedures.* Upper pictures* Allura Red dye injection into the renal artery.* Medial pictures* immunohistochemic staining for Laminin.* Lower pictures* immunohistochemic staining for Fibronectin. After decellularization with 0.66% SDS/60 min (*left column*) vascular network remains mainly preserved, as the vascular leakage is greater after the harshest decellularization protocol with 1% SDS/120 min (*right column*). Stainings for Laminin and Fibronectin reveal that the general matrix architecture is mainly preserved, even after using the harshest decellularization protocol (1% SDS/120 min, right column). Representative pictures. *Black bars* indicate 100 µm. *n* = 3

Integrity oft the vascular system was investigated by injection of Allura Red dye into the renal artery. After injection, the viscous dye fills the vascular network, until it reaches the renal vein and flows out into the surrounding medium. Kidneys decellularized using 60-min perfusion with 0.66% SDS showed an intact vascular network. The Allura red dye remained within the vessels, until it reached the renal vein. In contrast, the harshest decellularization protocol (1% SDS/120 min) resulted in a greater vascular leakage. Here, Allura Red dye entered more into the interstitium, before it reached the renal vein (Fig. 3).

To assess the preservation of the general tissue architecture, decellularized kidneys were stained for Laminin and Fibronectin, two components of the basal membrane. Here, the main architecture of the extracellular matrix was preserved, even after decellularization using the harshest protocol, using 120-min perfusion with 1% SDS (Fig. 3).

### Toxicity testing

To characterize SDS elution from the biomatrix, SDS was detected in the washing solutions at the end of each washing step (washing 1–5; compare Table 1). The detection limit of the used testing was 0.0025% SDS. Washing solutions from kidneys decellularized with 0.25% SDS/30 min, 0.5% SDS/60 min, 0.66% SDS/60 min, and 1% SDS/120 min were probed. In none of the experiments, SDS was detectable (data not shown).

**Fig. 4:**
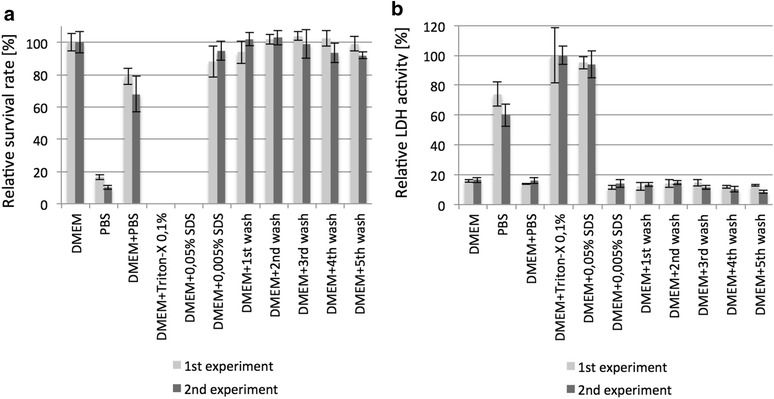
Toxicity testing of the rinsing solutions with C2C12-cells. **a** Alamar blue testing of relative cell survival. **b** LDH testing to detect cell death. The testing shows that no flowout of the washing steps contains SDS in concentrations inducing toxicity to the indicator cells. DMEM: cells cultivated in 100% DMEM; PBS: cells cultivated in 100% PBS; DMEM + PBS: cells cultivated in 1:1 mixture of DMEM and PBS; DMEM + SDS and DMEM + Triton-X: 1:1 mixture of DMEM and SDS, respectively, Triton X-100 in given concentration in PBS; DMEM + washing: 1:1 mixture of DMEM and collected flowout from the certain washing step

In addition, washing solutions from the experiments mentioned above were tested by indicator cells to detect toxic effects. As we are mainly interested in musculoskeletal tissue engineering, we used the murine C2C12-cell line. Here, none of the washing solutions showed toxicity in the cell assay. Figure 4 shows the results of the harshest protocol employing decellularization with 1% SDS for 120 min (Fig. 4).

**Fig. 5:**
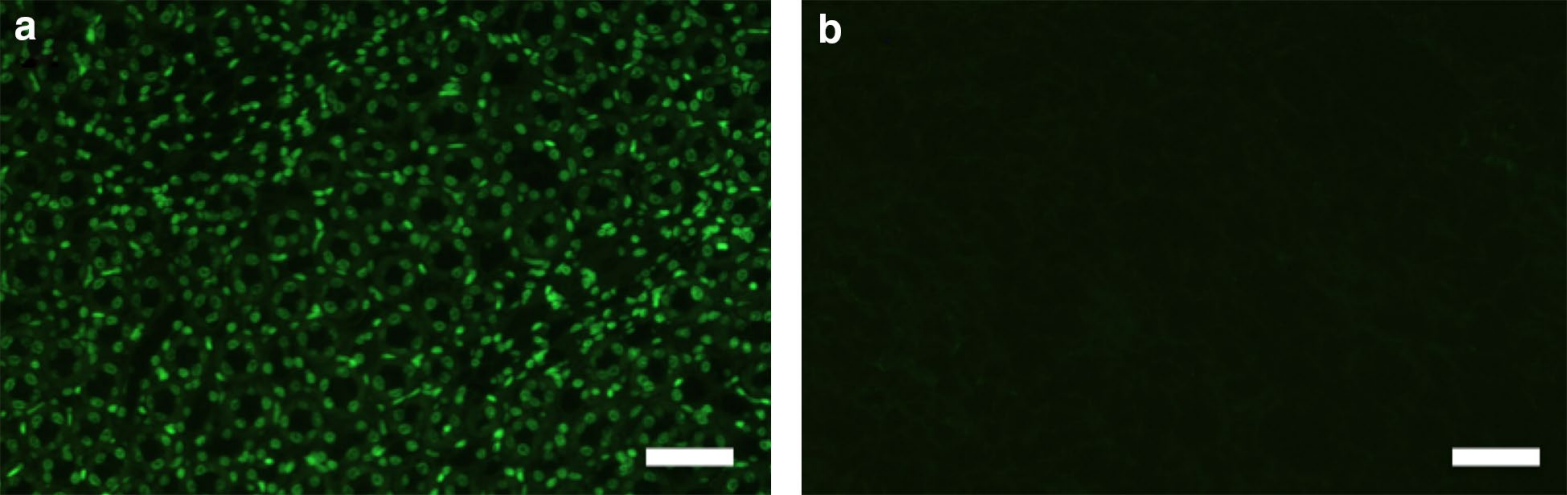
DNA detection by SybrGreen staining. **a** Native kidneys; **b** Kidneys decellularized with SDS (0.66%, 60 min). Representative pictures. *Scale bars* indicate 100 µm. n = 3

**Fig. 6:**
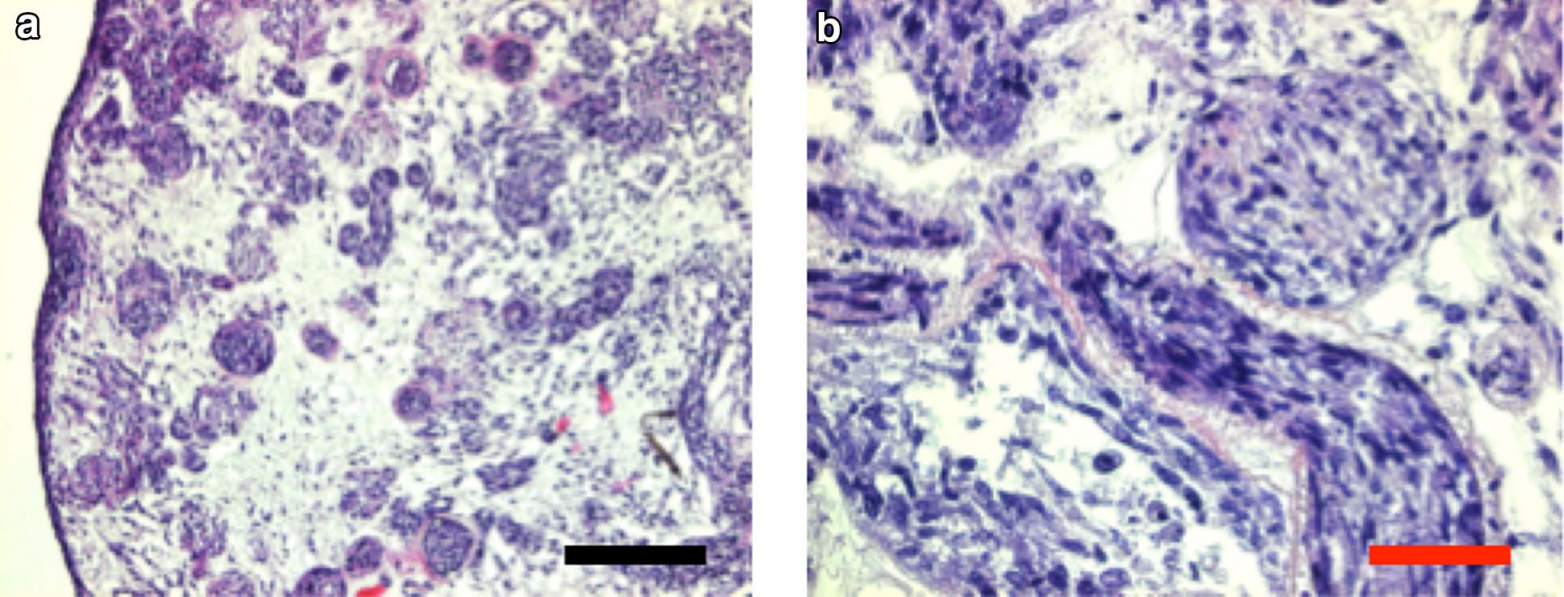
Histology at 5 days after seeding primary human osteoblasts into the decellularized kidneys. Decellularization was performed with 0.66% SDS for 60 min. **a** Overview magnification 10×. **b** Detailed section magnification 32×. Histology reveals no signs for matrix toxicity. After 5 days of culture, cells are growing in large areas of the matrix. *Cell shape* appears normal without signs of apoptosis or necrosis. Representative pictures. *Black bar* indicate 100 µm, *Red bar* indicate 30 µm. *n* = 3

To further investigate cell toxicity of the matrix, we probed kidney matrices decellularized with the most promising protocol, including 60-min decellularization with 0.66% SDS. First, we performed Sybr Green staining to investigate for assessing the remaining DNA. Here, the sensitive staining detected no DNA after the decellularization procedure (Fig. 5). Furthermore, we investigated direct cell toxicity of the matrix by seeding cells into the matrix. Here, we used primary human osteoblasts, as our group is focusing on musculoskeletal tissue engineering. Cells were seeded into the decellularized kidneys and cultivated under dynamic conditions for 5 days. This experiment was also performed in rat kidneys decellularized with 0.66% SDS for 60 min. After the cultivation period, cell survival rate was investigated by histology. At day 5, cells were present in large areas of the whole matrix. Histology revealed normal cell shape without signs of necrosis or apoptosis (Fig. 6).

## Discussion

In the present study, we established a standardized, time--efficient, and reproducible protocol for solid tissue decellularization. The procedure provides a complete decellularized extracellular matrix with preservation of the general tissue architecture, including the inherent vascular network. Our study confirms that this matrix is capable of growing cells under dynamic conditions without signs of remaining cell toxicity. This technique can serve as a platform technology for tissue engineering of vascularized tissues, as it can provide a solution to solve the existing limiting problem of vascularization.

A number of agents were characterized for their possible applicability on tissue decellularization. These are physical, chemical, and enzymatic agents [9]. The advantage of the physical decellularization methods, such as freezing and thawing, is their uniformly equal effect on the whole tissue sample, regardless of diffusion or perfusion. This makes the effect of physical methods to be more predictable in comparison to chemical or enzymatic agents [9]. However, the physical methods lead to effective cell lysis, but the cell remnants are not removed from the matrix, where they can cause unwanted immunologic or biological effects. Thus, physical cell lysis has to be combined with chemical or enzymatic methods to clear the matrix free of cellular debris [10]. As the enzymatic agents lead to less-reproducible clearance results and induce the risk of significant changes in the extracellular matrix composition, we decided to use a chemical agent as an alternate decellularization and clearance step [10]. Here, usually nonionic, ionic, or zwitterionic detergents are used. Of these, the ionic detergents are the most effective in solubilizing cytoplasmic membranes, lipids, and DNA. However, their use induces the risk of denaturation of the matrix proteins and thereby causes disruption to the integrity of the matrix, when used in higher concentrations [11]. Nonetheless, a mild denaturation of proteins can be favorable, as it eliminates antigens and thus reduces the risk for immunogenic adverse effects [7]. We used the ionic detergent SDS for chemical decellularization, as other studies had revealed that it is the most effective detergent in comparison to deoxycholate, Triton X-100, and peracetic acid [12].

The established decellularization procedure combines both physical and chemical decellularization procedures. This combination of different decellularization agents is favorable, as it increases efficacy and safety of the decellularization procedure [13]. Furthermore, it allows reducing detergent concentration and exposure time of the chemical decellularization. Thereby, it reduces damage inflicted to the extracellular matrix [14, 15].

Several other groups have shown successful decellularization of solid organs using detergents [3–5, 13, 16, 17]. For decellularization, we used detergent perfusion, as immersion of a solid tissue only leads to decellularization of tissues up to 5-mm thickness [18].

The established protocol possesses the combined advantages of the shortest perfusion time and the lowest SDS concentration, which lead to reliable tissue decellularization. The detergent perfusion lasts only 1 h. In total, the whole process lasts only 7 h, which is much shorter than all the other previously established methods, lasting between 1 and 10 days [3, 4, 16, 17, 19]. Even a previously published, time-optimized decellularization protocol includes four times longer SDS perfusion [20]. The sufficient amount of decellularization of our procedure after only 60 min is likely due to the impact of the additional physical cell lysis by freezing and thawing after harvesting.

Furthermore, most protocols include recircularization of the detergent solution. During our experiments, we demonstrated that avoiding recircularization and using fresh detergent solution resulted in marked increase in the decellularization efficacy (data not shown).

On investigating the kinetics of protein elution from the scaffolds, we found that this is dependent on the SDS concentration. At SDS concentrations of 0.66% and more, protein levels within the detergent flowout were almost undetectable after 30 min, indicating that by far the major process of decellularization is completed after 30 min. This kinetics is markedly different from the findings published by Wang et al. That author group found the decline only after 6 h, even when performing perfusion with 1% SDS [12]. This is likely due to the different organ size; whereas we used kidneys from rats, those authors used kidneys from mini pigs. This underlines the fact that the size of the tissue critically influences the kinetics of the decellularization process.

However, SDS is relatively a strong detergent leading to matrix degeneration, if used in high concentrations and subjected to long exposure time [14, 15]. Our study revealed preservation of the matrix architecture with the conservation of the basal membrane, after the established decellularization procedure. However, with high SDS concentrations of 1%, we observed significantly greater leakage of the vascular network. To avoid this with regard to later perfusion and revascularization of the decellularized matrix, we used the lowest SDS concentration, leading to safe decellularization for the established protocol.

It is known that SDS remaining in the decellularized tissue is toxic for cells reseeded into the matrix [21, 22]. The washing in all tested protocols seemed to be sufficient, as we were neither able to detect remaining SDS nor SDS-related cytotoxicity within the washing solutions. Furthermore, cells seeded into the decellularized matrix showed good survival rate without histologic signs for cytotoxity even after 5 days of growing.

Furthermore, we could demonstrate that the matrix obtained from rat kidneys is sufficient to grow cells from other species and tissue origin within. This underlines the potential of the established platform technology to generate a universal tissue engineering matrix for vascularized tissue engineering.

However, the present study possesses some limitations. The established protocol is optimized for decellularization of rat kidneys. It is likely that decellularization kinetics is different in other tissue sources and needs further adaption of the protocol proposed in this study. We investigated the applicability of the obtained matrix to grow cells from other tissues and species origin by cultivating human osteoblasts within. Of course, other cell types could be more sensitive to the matrix environment. Therefore, further studies with different cell types and decellularized matrices from different tissues and species are necessary.
